# Competition of three species of *Sitophilus* on rice and maize

**DOI:** 10.1371/journal.pone.0173377

**Published:** 2017-03-06

**Authors:** Christos G. Athanassiou, Nickolas G. Kavallieratos, James F. Campbell

**Affiliations:** 1 Laboratory of Entomology and Agricultural Zoology, Department of Agriculture Crop Production and Rural Development, University of Thessaly, Nea Ionia, Magnissia, Greece; 2 United States Department of Agriculture (USDA), Agricultural Research Service (ARS), Center for Grain and Animal Health Research (CGAHR), Manhattan Kansas, United States of America; 3 Laboratory of Agricultural Zoology and Entomology, Department of Crop Science, Agricultural University, Athens, Attica, Greece; Universidade Federal de Vicosa, BRAZIL

## Abstract

Laboratory tests were carried out in order to examine competition among three congeneric species on rice and maize: the granary weevil, *Sitophilus granarius*, the rice weevil, *Sitophilus oryzae* and the maize weevil, *Sitophilus zeamais*. For this purpose, a total of 30 adults were placed in vials that contained 50 g or either rice or maize: 30 adults of *S*. *granarius*, 30 adults of *S*. *oryzae*, 30 adults of *S*. *zeamais*, 15 adults of *S*. *granarius*+15 adults of *S*. *oryzae*, 15 adults of *S*. *granarius*+15 adults of *S*. *zeamais*, 15 adults of *S*. *oryzae* +15 adults of *S*. *zeamais*, and 10 adults of *S*. *granarius*+10 adults of *S*. *oryzae*+10 adults of *S*. *zeamais*. After 62 days at 30°C and 65% relative humidity the number of individuals of each species were counted. Insect damaged kernels (IDK), weight of frass and grain weight were measured. When each species was alone, *S*. *granarius* had the lowest numbers of adults in both grains, which did not exceed 34 adults/vial, and *S*. *oryzae* numbers were always higher than other species. For *S*. *oryzae* and *S*. *zeamais*, the numbers of adults were considerably higher on rice than on maize. On rice, *S*. *oryzae* numbers ranged between 281 and 563 adults per vial, while for *S*. *zeamais* between 137 and 372 adults per vial. At the same time, for both species on maize, adult numbers did not exceed 54 adults per vial. The number of *S*. *oryzae* adults were constantly higher than the other species in all combinations tested. Moreover, for rice, IDK in the vials that contained *S*. *oryzae*, either alone or in combination with other species, was higher than all the other combinations. Similarly, grain weight was lower in the vials that contained *S*. *oryzae* compared to the other species combinations. In general, for *S*. *oryzae* and *S*. *zeamais* progeny production was increased with the increase of the number of the initial adults that had been placed inside the vials. At the same time, progeny production of all three species was not affected by the presence of another species inside the vial. Given that the coexistence of congeneric species in the same stored product ecosystem is often reported, our results highlight some of the inferences that are necessary in order to predict the potential outcome of competition patterns. Apart from its ecological significance, the prediction of the superior species in mixed species communities, can guide and time any control measures, on a more species-targeted basis.

## Introduction

The coexistence of different stored product insect species at the same time with a given grain storage facility can be due to their roles in ecological succession and a direct consequence of differences in the way that each insect infests the commodity. Primary colonizers are able to exploit intact grain kernels, secondary colonizers exploit broken kernels or kernels already damaged by primary colonizers, and fungus feeders exploit grain that has undergone further deterioration [1,2]. In a grain storage structure or a facility that contains whole kernels of grain, colonization is expected to start with primary colonizers, whose adult feeding damage and immature stage development within grain kernels, increases the suitability of the grain to infestation by species with different trophic functions [2]. It is commonly reported that different species of secondary colonizers occur together in grain, but it is less common to find co-occurrence of different primary colonizing species [3,4]. For example, Nansen et al. [5] conducted sampling in a maize facility for 13 consecutive weeks, and found high densities of secondary colonizers such as the red flour beetle, *Tribolium castaneum*, the rusty grain beetle, *Cryptolestes ferrugineus* and the foreign grain beetle, *Ahasverus advena*, but only one primary colonizer, the maize weevil, *Sitophilus zeamais*. These observations indicate that inter-specific competition of primary stored product insect species may be stronger than that for secondary insect species. However, a number of other studies have found different species of primary colonizers co-occurring in grain [6–11]. For example, Buchelos and Athanassiou [8] in a horizontal facility with stored barley, recorded that the rice weevil, *Sitophilus oryzae* and the lesser grain borer, *Rhyzopertha dominica*, co-occurred during an one-year sampling period. However, direct competition may be avoided if there is finer scale spatial separation in the grain mass, and typically the sampling protocols used in the studies reported above are not fine enough so that this could be evaluated [10].

Competition of stored product insects, especially in beetle species, has been thoroughly examined in numerous publications. In fact, stored product beetles have been used extensively as model species to shape the theoretical background regarding the fundamentals of the ecology of competition. Crombie [12] showed how primary and secondary colonizers can coexist for a long time by evaluating the simultaneous presence of *R*. *dominica* and the sawtoothed grain beetle, *Oryzaephilus surinamensis* under laboratory conditions. Birch [13] examined the competition between two primary colonizers, *R*. *dominica* and *S*. *oryzae*, at different temperature and moisture content levels and found that the superior competitor was influenced by both variables. For example, on wheat with 14% moisture content, *R*. *dominica* was the superior competitor at 32°C, while *S*. *oryzae* was the superior competitor at 29°C. Giga and Canhao [14] found that on maize, *S*. *zeamais* can outcompete the larger grain borer, *Prostephanus trunctatus* at 25°C, but the outcome of this competition is uncertain at 30°C. All these studies documented that competition that leads to extinction is extremely common in primary insects.

Although primary colonizers can all exploit intact kernels of grain, there is variation in the strategies used to infest the grain kernel. For example, the females of *R*. *dominica*, oviposit externally to the kernel and newly hatched larvae chew into the kernel with all subsequent development completed inside the kernel, while, in contrast, females of *S*. *oryzae*, *S*. *zeamais* and the granary weevil, *Sitophilus granarius*, chew a hole into the kernel and oviposit directly inside the kernel [15–17]. These adaptation characteristics give certain advantages in competition outcome, but there are disproportionally few studies on the competition of primary colonizers that belong to the same genus, using similar strategies to lay eggs, develop and infest the commodity. Due to similarities in infestation patterns, competition may be expressed more vigorously in the case of relative species than on primary colonizers that belong to different genera. For example, in a competition study, Giga and Smith [18] found that the hatchability of eggs of the southern cowpea weevil, *Callosobruchus maculatus* was significantly reduced compared with the adzuki bean weevil, *Callosobruchus rhodesianus* as the adult density increases.

There are very few data regarding competition among the species of the genus *Sitophilus*, although there are several reports that show coexistence in storage facilities. Athanassiou and Buchelos [19] noted the coexistence of *S*. *zeamais* with either *S*. *oryzae* or *S*. *granarius* in the same sample was not very common, but the coexistence of *S*. *oryzae* and *S*. *granarius* was common. Moreover, the type of commodity appeared to influence *Sitophilus* species composition within a sample, perhaps due to its influence on basic population growth parameters. Gökçe [20] noted that in laboratory bioassays using *S*. *oryzae* and *S*. *granarius*, the former species was always dominant, but this level of dominance was highly moderated by the type of product. Still, to our knowledge, there are no data generated using the same protocols available on competition among the three *Sitophilus* species typically found infesting grain. In the present study, we examined, under laboratory conditions, the coexistence patterns of *S*. *granarius*, *S*. *oryzae* and *S*. *zeamais* on rice and maize.

## Materials and methods

### Insects and commodities

*Sitophilus granarius* and *S*. *oryzae* were reared on whole wheat kernels, while *S*. *zeamais* was reared on whole maize kernels, at 27°C and 65% relative humidity. Both species were maintained in culture at CGAHR for more than 30 years. Adults, less than 2 weeks-old, were used in the tests. Untreated, clean, rice (mixture of varieties) and maize (mixture of varieties) used in the experiments were held at subzero temperatures prior to the initiation of the experiments to eliminate any prior infestation. The rice and maize were warmed to room temperature and adjusted to 13% moisture content before the tests.

### Competition tests

Fifty grams of either rice or maize was placed in plastic vials (11 cm in height, 5.3 in diameter). Then, a total of 30 weevils were introduced into each vial, in the following combinations: 30 *S*. *granarius*, 30 *S*. *oryzae*, 30 *S*. *zeamais*, 15 *S*. *granarius*+15 *S*. *oryzae*, 15 *S*. *granarius*+15 *S*. *zeamais*, 15 *S*. *oryzae*+15 *S*. *zeamais* and 10 *S*. *granarius*+10 *S*. *oryzae*+10 *S*. *zeamais*. All vials were placed in an incubator chamber set at 30°C, 65% relative humidity and continuous darkness. For each species-grain combination, there were nine replicate vials for each treatment, performed in three blocks of three replicates each. After 62 days of having insects in the grains, all vials were removed from the chamber and placed in a freezer at -5°C for 7 days to kill all insects, and then the adults in each vial were identified to species and counted. The number of insect damaged kernels (IDK) (i.e., kernels with exit holes and/ or chewings) per sample of 30 kernels per vial was also recorded. The weight of all kernels within each vial, as well as weight of the frass and fine materials, were also measured. The weight of kernels was determined after sieving to remove frass and fine materials.

### Data analysis

The data for insect counts were submitted to a two-way ANOVA with weevil species and grain type as main effects, with weevil counts as the response variable. For IDK, frass production and grain weight, two-way ANOVA was used, with number of IDK, weight (g) of frass and weight of grains (g) without the frass, as the response variables, with commodity and weevil species as main effects. The associated interactions of the main effects were also incorporated in the analyses. For analysis of grain characteristics, we excluded vials where grain was so heavily damaged that fungal growth due to high moisture was excessive. All analyses were conducted using the JMP 11 software (SAS Institute Inc., Cary, NC, U.S.A.). Means were separated by using the Tukey-Kramer HSD test at α = 0.05.

## Results

### Insect counts

For *S*. *granarius*, both main effects were significant, but the interaction was not (Table 1). On rice, the largest number of *S*. *granarius* adults occurred when it was the only species present, although the mean number was only four individuals more than the starting number so reproduction was limited (Table 2). When combined with *S*. *oryzae* and *S*. *zeamais*, significantly fewer individuals were recovered than when reared alone. Interestingly, although the total numbers of *S*. *granarius* were lower, the proportional increase in numbers was greater than when only 30 *S*. *granarius* were added (~2 fold increase). On maize, significantly more *S*. *granarius* adults were found in the vials that contained only *S*. *granarius*, as compared with the other combinations (Table 2). Unlike on rice, the numbers of individuals at the end of the study was similar to the number added for all species combinations. When alone or with *S*. *zeamais*, more progeny were produced by *S*. *granarius* on rice than on maize.

**Table 1:** ANOVA parameters for comparison of adult numbers in vials at end of the test among species and grain type (rice or maize).

		Weevil species					
		*S*. *granarius*		*S*. *oryzae*		*S*. *zeamais*	
Source	df	F	p	F	p	F	p
Species	3	16.6	<0.01	9.1	<0.01	4.6	<0.01
Grain	1	18.6	<0.01	215.8	<0.01	56.1	<0.01
Species x Grain	3	0.7	0.54	6.4	<0.01	4.0	0.01

In all cases, total df = 71.

**Table 2:** Mean adult number of *S*. *granarius*, *S*. *oryzae* and *S*. *zeamais* adults ± SE per vial on rice after 62 days.

	Final adult number per vial for each commodity					
	Rice			Maize		
Initial adult number for each species in vial	*S*. *granarius*	*S*. *oryzae*	*S*. *zeamais*	*S*. *granarius*	*S*. *oryzae*	*S*. *zeamais*
30 *S*. *granarius*	34.0±1.1 aA			30.6±0.5 aB		
30 *S*. *oryzae*		563.4±43.0 aA			53.4±4.1 aB	
30 *S*. *zeamais*			371.9±27.4 aA			30.8±1.8 aB
15 *S*. *granarius* + 15 *S*. *oryzae*	24.6±4.2 abA	331.7±59.8 bA		15.9±0.5 bcA	38.0±4.9 abB	
15 *S*. *oryzae* + 15 S. *zeamais*		326.7±36.9 bA	239.1±96.2 abA		33.3±3.8 bB	25.2±2.0 abB
15 *S*. *zeamais* + 15 *S*. *granarius*	27.9±2.5 abA		145.1±26.4 bA	18.2±0.8 bB		27.0±2.8 abB
10 of each species	20.0±3.5 bA	280.7±39.9 bA	136.8±20.1 bA	13.7±1.9 cA	26.2±4.4 bB	18.8±2.6 bB

Within each column, means followed by the same lowercase letter are not significantly different (F values ranged between 3.7 and 47.6, p values ranged between between 0.02 and <0.01, Tukey-Kramer HSD test at p = 0.05; in all cases df = 3, 32). Within each row, means followed by the same uppercase letter are not significantly different (F values ranged between 2.1 and 154.4; p values ranged between 0.16 and <0.01, Tuckey-Kramer HSD test at p = 0.05; in all cases df = 1, 16).

For *S*. *oryzae*, both main effects and their interaction were significant (Table 1), with the number of *S*. *oryzae* being greater on rice than maize, and also tending to be reduced when combined with other species (Table 2). On rice, progeny production was greatest when 30 *S*. *oryzae* were added alone, with a 19 fold increase from initial number. There were no significant differences in *S*. *oryzae* progeny production when paired with either *S*. *granarius or S*. *zeamais*, individually or all three species combined. Even though the total number of progeny was reduced, the proportion increase was similar or greater (22 fold increase in pairs and 28 fold increase when three species present) to that obtained when 30 individuals of the same species were added. On maize, progeny production of *S*. *oryzae* was significantly lower than that on rice for all treatments: only a 2-fold increase when alone and increases ranging between 2.2 and 2.6 fold. Significantly more *S*. *oryzae* adults were recorded when this species was alone than in any other treatment, except when combined with *S*. *granarius* where progeny number was not different from any of the other treatment combinations (Table 2).

For *S*. *zeamais* both main effects and their interaction were significant (Table 1), and number of progeny was greater on rice than on maize for all combinations tested (Table 2). On rice, progeny production was greatest when this species was alone and lowest when combined with *S*. *granarius* or when all three species were present, but the combination of *S*. *oryzae* and *S*. *zeamais* was not different from any of the treatment combinations. When alone there was a 12-fold increase and when combined with other species the increase ranged between 10 and 16 fold. On maize, progeny production was similar in all treatments, except that progeny production was greater when *S*. *zeamais* was alone compared to when three species were present (Table 2). There was no increase in numbers when 30 *S*. *zeamais* were held on maize, but when in combination with other species the increase was about 2 fold.

### Grain damage

For IDK, frass, and grain weight, both main effects and their interactions were significant (Table 3). Mean numbers of IDK were greater on rice than on maize for all species either held individually or in combination (Tables 4–6). Similarly, significantly more amount of frass was produced on rice than on maize in all cases except when *S*. *oryzae* was combined with *S*. *zeamais* (Table 5). After removing frass, mean rice weight was significantly lower than maize either when the three species were kept alone or in any combination (Table 6). Overall, *S*. *oryzae* caused the greatest amount of damage, increased IDK and frass and decreased grain weight, and *S*. *granarius* caused the least amount of damage.

**Table 3:** ANOVA parameters for insect damaged kernels on vials with different species combination and different grains.

		Insect damaged kernels		Frass		Grain weight	
Source	df	F	p	F	p	F	p
Species combination	6	65.0	<0.01	27.1	<0.01	43.7	<0.01
Grain	1	655.6	<0.01	156.4	<0.01	496.6	<0.01
Species combination x Grain	6	4.2	<0.01	14.2	<0.01	24.4	<0.01

For insect damaged kernels, total df = 112. For frass and grain weight, total df = 106.

**Table 4:** Mean number of IDK ± SE per vial in a sub-sample of 30 kernels for vials containing different combinations of *S*. *granarius*, *S*. *oryzae* and *S*. *zeamais* on rice or maize.

Species and number of adults in the vial	Rice	Maize	F	p
30 *S*. *granarius*	21.3±1.1 bA	8.4±0.8 bcB	85.2	<0.01
30 *S*. *oryzae*	30.0±0.0 aA	12.1±1.5 abcB	143.2	<0.01
30 *S*. *zeamais*	19.8±1.4 bA	6.8±4.4 cB	61.1	<0.01
15 *S*. *granarius* + 15 *S*. *oryzae*	29.9±0.1 aA	13.1±1.6 abB	111.5	<0.01
15 *S*. *granarius* + 15 *S*. *zeamais*	20.5±1.2 bA	11.7±1.1 abcB	30.5	<0.01
15 *S*. *oryzae* + *S*. 15 *zeamais*	30.0±0.0 aA	14.3±1.6 aB	100.4	<0.01
10 of each species	30.0±0.0 aA	14.9±0.9 aB	279.2	<0.01
F	38.9	5.9		
p	<0.01	<0.01		

Within each column, means followed by the same lowercase letter are not significantly different (Tuckey-Kramer HSD test at p = 0.05; in all cases df = 6, 56). Within each row, means followed by the same uppercase letter are not significantly different (Tukey-Kramer HSD test at p = 0.05; in all cases df = 1, 16).

**Table 5:** Mean weight (g) ± SE per vial of frass in vials containing different combinations of *S*. *granarius*, *S*. *oryzae* and *S*. *zeamais* on rice or maize.

Species and number of adults in the vial	Rice	Maize	F	p
30 *S*. *granarius*	0.32±0.03 dA	0.24±0.02 cB	5.3	0.04
30 *S*. *oryzae*	2.41±0.69 aA	0.66±0.06 abB	21.9	<0.01
30 *S*. *zeamais*	1.35±0.12 bcA	0.24±0.03 cB	80.5	<0.01
15 *S*. *granarius* + 15 *S*. *oryzae*	1.87±0.17 abA	0.62±0.06 abB	59.2	<0.01
15 *S*. *granarius* + *S*. 15 *zeamais*	0.89±0.15 cdA	0.47±0.04 abB	7.2	0.02
15 *S*. *oryzae* + *S*. 15 *zeamais*	0.60±0.15 cdA	0.64±0.06 bA	0.1	0.76
10 of each species	1.76±0.17 abA	0.70±0.07 aB	48.7	<0.01
F	15.1	14.1		
p	<0.01	<0.01		

Within each column, means followed by the same lowercase letter are not significantly different (Tuckey-Kramer HSD test at p = 0.05; for rice df = 6, 37, for maize df = 6, 56). Within each row, means followed by the same uppercase letter are not significantly different (Tukey-Kramer HSD test at p = 0.05; df ranged between 1, 10 and 1, 16).

**Table 6:** Mean grain weight (g) ± SE per vial of the kernels without the frass in vials containing different combinations of *S*. *granarius*, *S*. *oryzae* and *S*. *zeamais* on rice or maize.

Species and number of adults in the vial	Rice	Maize	F	p
30 *S*. *granarius*	35.89±0.15 aA	37.88±0.16 aB	82.0	<0.01
30 *S*. *oryzae*	17.46±5.04 cΑ	35.11±0.39 bB	43.0	<0.01
30 *S*. *zeamais*	28.47±0.72 bA	37.65±0.20 aB	148.7	<0.01
15 *S*. *granarius* + 15 *S*. *oryzae*	21.91±1.33 cA	35.01±0.36 bB	103.3	<0.01
15 *S*. *granarius* + *S*. 15 *zeamais*	32.78±0.63 abA	36.44±0.31 abB	27.2	<0.01
15 *S*. *oryzae* + *S*. 15 *zeamais*	18.60±3.62 cA	35.43±0.43 bB	68.9	<0.01
10 of each species	20.21±2.58 cA	35.35±0.41 bB	75.8	<0.01
F	24.9	12.8		
p	<0.01	<0.01		

Within each column, means followed by the same lowercase letter are not significantly different (Tuckey-Kramer HSD test at p = 0.05; for rice df = 6, 37, for maize df = 6, 56). Within each row, means followed by the same uppercase letter are not significantly different (Tukey-Kramer HSD test at p = 0.05; df ranged between 1, 10 and 1, 16).

On rice, the number of IDK was significantly higher in all combinations that contained *S*. *oryzae* (~30 IDK out of 30 kernel sub-sample), as compared with the combinations that did not contain *S*. *oryzae* (Table 4). This suggests that reproduction on rice may have been limited for this species because all the kernels had been exploited. On rice, frass was generally greater when *S*. *oryzae* and *S*. *zeamais* were present, with the exception of *S*. *oryzae*+*S*. *zeamais* (Table 5). In contrast, the lowest amount of frass was recorded in vials that contained *S*. *granarius* alone. Grain weight was significantly lower in all combinations with *S*. *oryzae*, as compared to the species combinations that did not contain *S*. *oryzae* (Table 6). In these vials, the grain weight ranged between 17–22 g per vial, which was less than one half of the initial grain weight. In contrast, the highest grain weight was recorded in vials that contained *S*. *granarius* alone (22% reduction in weight), followed by those that contained *S*. *granarius*+*S*. *zeamais* and *S*. *zeamais* alone.

With maize, the number of IDK did not exceed 15 IDK out of 30 kernels and there were limited significant differences among the species combinations (Table 4). Frass weight did not exceed 0.7 g per vial for any of the species combinations evaluated using maize. In this commodity, the lowest amount of frass was noted in vials that contained either *S*. *granarius* or *S*. *zeamais* alone (Table 5). The greatest weight reduction was noted in any of the species combinations containing *S*. *oryzae* (ranging between 29 and 30%), except for the combination of *S*. *granarius* and *S*. *zeamais* that was not different from any of the species combinations (Table 6).

## Discussion

To our knowledge, this is the first report in which competition among the three *Sitophilus* species was examined. The species differed in their reproductive rate which impacted competitive outcomes, but this was moderated by the type of grain. For all three species, regardless of the treatment combinations, rice was much more suitable for progeny production than maize. This was more evident for *S*. *oryzae* and *S*. *zeamais*, where weevil emergence was often 10 fold or greater on rice than on maize. This highlights that although the common names for these species are associated with specific grains, that does not mean that these grains are the most suitable for development. In contrast, *S*. *granarius* had lower progeny production on both commodities, although progeny production was slightly greater on rice than on maize.

Differences in biological parameters and life table characteristics are, apparently, responsible for the dissimilar population growth patterns recorded here for the three species tested. Based on our data, *S*. *granarius* had the lowest numbers from the three weevil species tested, regardless of the type of the commodity and the species combination examined. When held alone there was essentially no population increase on both maize and rice. While the comparable studies of life table characteristics of the three weevil species are very few, it is known that mated *S*. *granarius* adult females can lay ~200 eggs [21], while oviposition of the other species can reach ~400 eggs per female [21,22], which is consistent with the results of our study although the progeny production was much less than half that of the other species. Also, *S*. *granarius* has a slower developmental rate in comparison with the other two *Sitophilus* species [15]. For example, at 25°C *S*. *granarius* needs 45 days for egg to adult development, while for *S*. *oryzae* and *S*. *zeamais* this interval is 40 days or shorter. This difference between *S*. *granarius* and the other species is wider with the increase of temperature to 30°C [15,21]. It is interesting that *S*. *zeamais* did better when in some of the species combinations than when alone, but it is unclear if this is a shift in the number of eggs laid or development rate and if this is due to some change in the grain characteristics or some recognition of the beetles of other weevil species presence or lower density of conspecifics.

Typically only one individual will develop to adulthood and emerge from a kernel in cases where more than one egg is laid per kernel [15,23–26]. Although we did not measure immature developmental rates, the faster development of *S*. *oryzae* and *S*. *zeamais* larvae could lead to larger larvae of those species competing with smaller *S*. *granarius* larvae. When two tunneling larvae encounter each other in a kernel the larger larva typically kills the smaller individual [27]. Additional studies would be needed to evaluate the relative contribution of larval competition versus reduced oviposition to explain the low numbers of *S*. *granarius* adults recorded here.

Reduction in number of progeny produced by each species when paired with another species and when all three are together is partially due to reduction in the number of founding individuals added to the vials. However, differences between the grain types and differences in increase relative to number of founders does indicate that other factors are influencing progeny production. Commodity played a critical role. Both *S*. *oryzae* and *S*. *zeamais*, can be found in a variety of grains, and are known to be abundant on both maize and rice [16,21,24,28]. For storage facilities in Greece, Athanassiou and Buchelos [19] noted that *S*. *zeamais* was more frequently found on maize than on wheat or barley; however, in that study, the authors did not examine rice stores. Throne and Cline [29] monitored the seasonal abundance of *S*. *oryzae* and *S*. *zeamais* in storage facilities with different grains in South Carolina and noted that the latter species was found in greater numbers at all sites at the same periods of year, regardless of the type of the commodity that was stored. In light of our findings, maize can be considered as by far the less preferred commodity for *S*. *granarius*, since the number of the adults recorded at the end of the experimental period were close to the number of parental individuals, which means that the species did not produce progeny or progeny production was extremely limited. Historically, both *S*. *oryzae* and *S*. *zeamais* have been found to be associated with rice before the onset of agriculture, while maize-*S*. *zeamais* association was initiated more recently, i.e., after maize became a global crop [30,31]. Also, *S*. *zeamais* is generally more abundant than the other two *Sitophilus* species in warmer zones [31,32]. Laboratory studies such as ours can provide a clearer picture of host associations based on food suitability, which can be difficult to detect in field surveys where other factors such environmental conditions might also influence predominance of a species. For example, Cordeiro et al. [33] noted that the application of insecticides shifted the outcome of the competition between *S*. *zeamais* and *R*. *dominica*.

Regarding both *S*. *oryzae* and *S*. *zeamais*, at least at the range of the combinations tested here, progeny production of each species was not affected by the simultaneous presence of another species, and weevils that had emerged from vials that contained 30 parental adults were, with one exception, always more than the ones that had been emerged with vials with 10 or 15 parental adults of a given species. In contrast, there were no differences in progeny production counts between vials containing 15 and vials containing 10 parental adults. Also, Danho et al. [34] indicated that, under laboratory conditions, *S*. *zeamais* progeny emergence was analogous to the maize quantity. Earlier studies suggest that, under similar conditions, *S*. *oryzae* is able to produce more eggs than *S*. *zeamais* [15]. Hence, Birch [35,36] and Segrove [37] showed that *S*. *oryzae* lays approximately 33% more eggs than *S*. *zeamais*, which supports the results of the current study, regarding progeny production patterns of these two species. In fact, regarding *S*. *zeamais* adult numbers, there were no significant differences between vials that contained *S*. *zeamais* alone with those that contained both *S*. *zeamais* and *S*. *oryzae*. Based on the above, at least in the first stage of colonization, *S*. *oryzae* is more likely to become the dominant species, but at a later stage, under the influence of density-dependent conditions, such as moisture constraints due to elevated temperatures or dispersal potential, *S*. *zeamais* may be prevalent [15,29], a fact that is particular important since both species share the same resources. Due to the limited food sources used in our tests, the competition outcome should be directly related to life table characteristics and population growth parameters, rather than behavioral and dispersal responses, but the outcome of the competition might have been different in commercial storage facilities [29,38].

The more rapid rate of population growth of *S*. *oryzae* on rice was evident based on the damage that it caused to grain. Among the three species tested here, *S*. *oryzae* had the highest contribution to kernel damage. This is related with the life table characteristics mentioned above. Ryoo and Cho [39] tested a wide range of temperatures and found that *S*. *oryzae* egg-to-adult development was only 29 days at 28°C. Thus, we assume that the duration of our experiment was sufficient for this insect to complete two generations, which in total resulted on a high number of individuals and damaged kernels. However, on maize the number of damaged kernels was considerably lower. Still, on maize, increased IDK is not directly associated with increased progeny production, at least in the degree that this was found on rice. Furthermore, frass was considerably lower on maize than on rice, which is an additional indicator that rice was the preferred commodity.

In summary, our work showed that the three *Sitophilus* species differ in their ability to exploit rice and maize and this is likely to lead over time to one species becoming dominant. However, over the time of the experiment all species maintained similar rates of increase even when exposed to one or more other species. Based on our results, *S*. *granarius* should be considered as the inferior competitor and *S*. *oryzae* as the superior competitor, in a “scramble competition” [18,40]. When there are restrictions in food availability, as in the case of laboratory-based competition studies with stored-product insects, the outcome of the competition can be expressed more rapidly [11]. On the other hand, on maize, which is the less preferred commodity by all three tested *Sitophilus* species, the outcome of the competition may be more gradual or even uncertain. Although *S*. *zeamais* is considered as a serious pest of stored-maize [15], previous reports have documented that maize may not favor its development. For example, Nwosu et al. [41] found that progeny production of a Nigerian *S*. *zeamais* population ranged from 0 to 10 adult individuals as determined at 56 days post-infestation on 20 Nigerian maize varieties. Soderstrom and Wilbur [42] reported that *S*. *zeamais* population originating from Arkansas (USA) produced 11–52 offspring individuals on maize while its progeny production was 80–481 individuals on wheat, all cultured in Kansas. Reasons that moderate *S*. *zeamais* development on maize run to kernel hardness, morphology of the pericarp and lipid, protein, amylose, sugar or phenolic contents [43–46]. Kiritani [47] also showed that among geographical strains of *S*. *zeamais* there is considerable variation in the number of progeny produced on maize, rice and wheat. Therefore, our results correspond to the certain strains and commodities used and generalizations should be avoided. Additional experimental work is required at longer experimental intervals, in order to indicate if some species would become extinct due to competition or coexistence can be continued for prolonged periods. Apparently, the initial number of adults used here can be considered as the main parameter that determined progeny production and the concomitant grain damage.
